# Estimation of Leaf Area Index and Plant Area Index of a Submerged Macrophyte Canopy Using Digital Photography

**DOI:** 10.1371/journal.pone.0051034

**Published:** 2012-12-04

**Authors:** Dehua Zhao, Dong Xie, Hengjie Zhou, Hao Jiang, Shuqing An

**Affiliations:** Department of Biological Science and Technology, Nanjing University, Nanjing, People’s Republic of China; University of Nottingham, United Kingdom

## Abstract

Non-destructive estimation using digital cameras is a common approach for estimating leaf area index (LAI) of terrestrial vegetation. However, no attempt has been made so far to develop non-destructive approaches to LAI estimation for aquatic vegetation. Using the submerged plant species *Potamogeton malainus*, the objective of this study was to determine whether the gap fraction derived from vertical photographs could be used to estimate LAI of aquatic vegetation. Our results suggested that upward-oriented photographs taken from beneath the water surface were more suitable for distinguishing vegetation from other objects than were downward-oriented photographs taken from above the water surface. Exposure settings had a substantial influence on the identification of vegetation in upward-oriented photographs. Automatic exposure performed nearly as well as the optimal trial exposure, making it a good choice for operational convenience. Similar to terrestrial vegetation, our results suggested that photographs taken for the purpose of distinguishing gap fraction in aquatic vegetation should be taken under diffuse light conditions. Significant logarithmic relationships were observed between the vertical gap fraction derived from upward-oriented photographs and plant area index (PAI) and LAI derived from destructive harvesting. The model we developed to depict the relationship between PAI and gap fraction was similar to the modified theoretical Poisson model, with coefficients of 1.82 and 1.90 for our model and the theoretical model, respectively. This suggests that vertical upward-oriented photographs taken from below the water surface are a feasible alternative to destructive harvesting for estimating PAI and LAI for the submerged aquatic plant *Potamogeton malainus*.

## Introduction

Leaf area index (LAI), defined as half the total developed area of green leaves per unit horizontal surface area [1], is one of the most important parameters characterizing plant canopy structure, processes and functions. LAI correlates with the photosynthesis, respiration and transpiration of the plant canopy and thus the exchange of matter and energy between ecosystem and atmosphere [2]. Therefore, measurement of LAI is one of most common tasks in ecosystem surveys [3], [4].

Numerous approaches have been developed for measuring LAI, including destructive and non-destructive techniques [2], [5]. Because destructive methods tend to be very time-consuming and poorly representative of the plant canopy, development of non-destructive methods has increased greatly in recent years, with non-destructive methods being recommended for the measurement or monitoring of LAI through time and across large spatial areas. Many optical instruments have been developed specifically for non-destructive LAI measurement, including Tracing Radiation and Architecture of Canopies (TRAC) [3], SUNSCAN Canopy Analysis System [6], AccuPAR [7], DEMON [8], LAI-2000 Plant Canopy Analyzer [9] and hemispherical photography [5], [10], [11]. In addition, consumer-grade digital cameras have been adopted widely for estimation of LAI in recent years due to economical and convenience considerations [12]–[17]. Most of the non-destructive methods operate on a similar principle, i.e., the gap fraction of a canopy is determined primarily by LAI and leaf inclination [5]. When the leaf inclination of a canopy is known or varies little between samples, vertical gap fraction can be used to estimate LAI [13]. When the leaf inclination of a canopy is unknown, the gap fraction of multiple angles or a certain angle insensitive to leaf inclination can be used to estimate LAI [5].

While accurate LAI measurement is important in terrestrial ecosystem studies, it is also of primary importance when working with aquatic submerged vegetation, which functions not only as the primary vehicle for photosynthesis but also plays a critical role in other aquatic ecosystem functions such as regulation of the nutrient cycle, stabilization of sediments and slowing of water currents [18]–[20]. Currently, destructive sampling is virtually the only approach documented for field sampling of LAI for submerged vegetation and is typically calculated using shoot density and mass weight of leaves [18], [21]–[24]. However, the characteristics of underwater growth make it difficult to perform accurate quantitative sampling of submerged vegetation (i.e. sampling per unit sediment area), which is problematic for the application of destructive methods for measuring LAI of submerged vegetation.

Whether non-destructive methods based on photographs taken by digital cameras are also suitable for *in situ* LAI measurements of submerged vegetation is a timely and important question. The advantages associated with digital camera methods apply to their use in aquatic as well as terrestrial ecosystems; therefore, if non-destructive methods using digital cameras prove to be feasible, they will likely be adopted widely by researchers and managers of aquatic systems to acquire ground measurements of LAI for the monitoring of aquatic vegetation, development of aquatic ecosystem models, validation of aquatic vegetation remote sensing classifications, and other purposes [2], [5], [12]. However, the use of digital cameras for measuring LAI of submerged vegetation will face challenges. First, the strong light absorption characteristic of water will necessarily result in higher ratios of blue and red bands in photographs of aquatic as compared with terrestrial vegetation [25], and thus the algorithm developed to distinguish vegetation from other objects for terrestrial vegetation may not be applicable to aquatic vegetation. Second, the absorption, reflection and scattering of organic and inorganic particles in water [26]–[28] add further difficulties to the identification of aquatic species in photographs. Third, models for the derivation of LAI from gap fraction that were developed for terrestrial vegetation may not be applicable to aquatic vegetation because of the differences in morphology between aquatic and terrestrial plants [5], [12]. To our knowledge, no attempts have been made thus far to measure LAI of submerged vegetation using digital cameras.

The aim of this study was to develop a non-destructive approach based on digital photography for the measurement of LAI and PAI (plant area index) of submerged vegetation using a plant species (*Potamogeton malainus*) dominant in lakes along the middle and lower reaches of the Yangtze River in China [29], [30]. Specific objectives for accomplishing this goal were to (1) determine the optimal photographic parameters (i.e., photo orientation – upward vs. downward; exposure settings; sunlight conditions) for distinguishing vegetation from other pixels in photographs of aquatic vegetation in order to obtain accurate gap fractions for calculation of LAI and PAI, and (2) develop a quantitative model for predicting LAI and PAI using the gap fraction derived from the digital photographs and destructive measurement of LAI and PAI and compare the model thus developed with a theoretical model.

## Materials and Methods

### 2.1 Ethics Statement

No specific permits were required for the described field studies. The location studied is not privately-owned or protected in any way and the field studies did not involve endangered or protected species.

**Figure 1:**
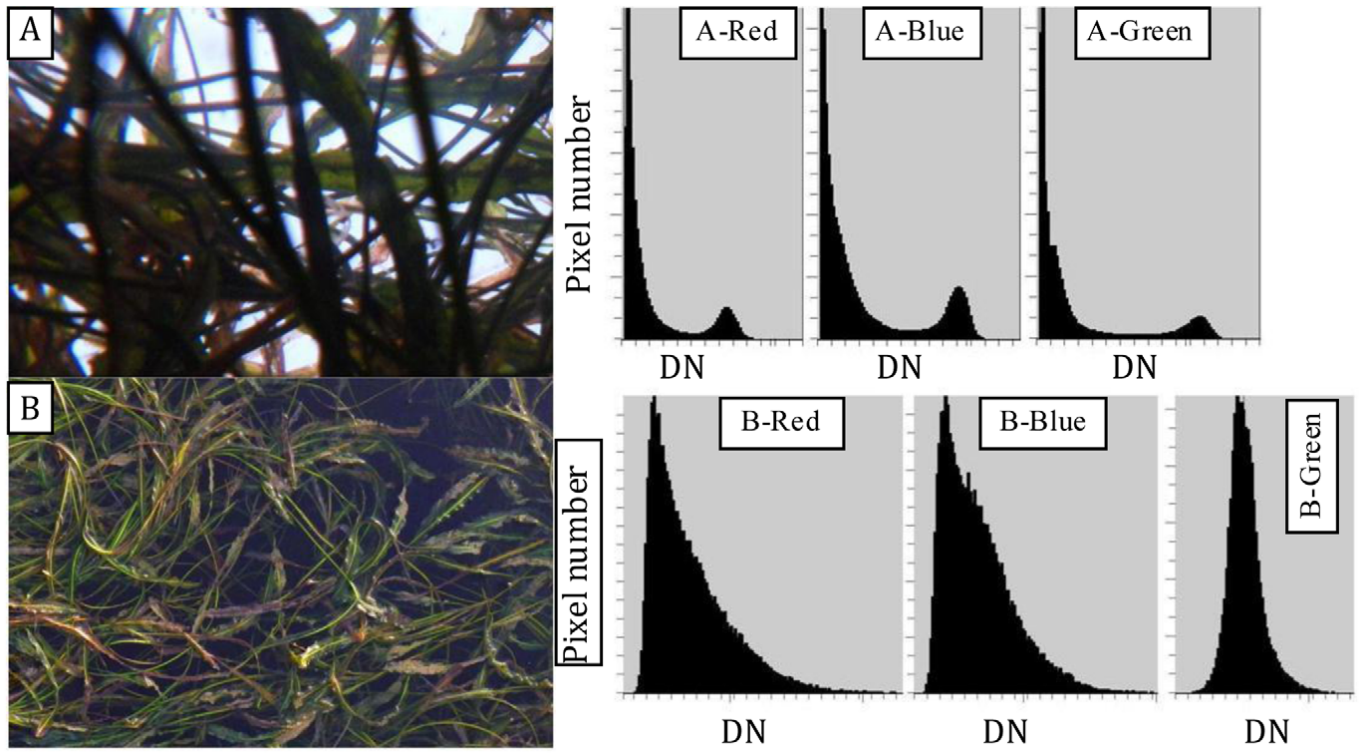
The influence of photograph orientation on digital number (DN) differences between vegetation and background pixels in vertical photographs of aquatic vegetation. A and B show vertical upward- and downward-oriented photographs, respectively. Histograms of DNs for red, blue and green bands are also shown for upward-oriented (A-Red, A-Blue and A-Green) and downward-oriented (B-Red, B-Blue and B-Green) photographs.

### 2.2 Site Description and Experimental Design

The study was conducted using 20 Fiber Reinforced Plastic (FRP) incubators (length = 1.7 m, width = 1.25 m, height = 1.0 m) placed along the shores of Taihu Lake, China (31°15′40″N, 120°0′57″). *Potamogeton malainus* was transplanted to the incubators on April 22–23, 2012. Before transplantation, sediment was salvaged from the southeastern part of Taihu Lake, where *Potamogeton malainus* was distributed naturally, and placed in the FRP incubators, forming a 5 cm silt layer. Total nitrogen (TN), total phosphorous (TP) and organic matter in the salvaged sediment were 0.113%, 0.099% and 1.37%, respectively. Next, water was pumped from Taihu Lake to completely fill the incubators, and full water status was maintained throughout the entire study period. TN, TP and total organic carbon (TOC) in the pumped water were 2.48, 0.17 and 7.79 mg/L, respectively. We produced high variability in LAI in the *Potamogeton malainus* canopy by establishing shoot densities varying from 20 to 200 plants/m^2^ in the incubators. Turbidity, which ranged from 2.21 to 4.36 NTU and averaged 3.32 NTU, was measured using a multi-parameter water quality checker (HORIBA, U-52) when photographs were taken and when the vegetation was harvested.

**Figure 2:**
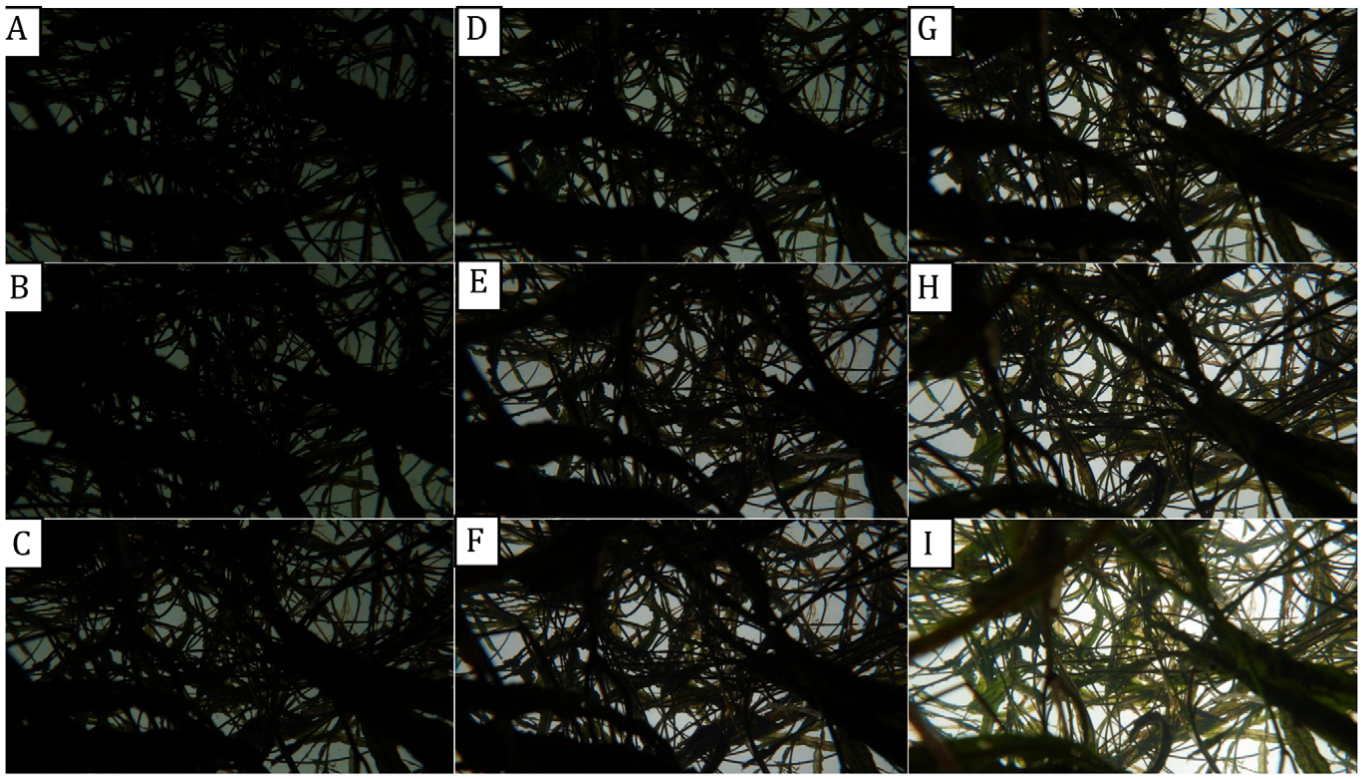
The influence of photographic exposure on digital number (DN) differences between vegetation and background pixels in vertical upward-oriented photographs. The aperture was fixed at F4.2. From A to I, the photographs were taken with shutter speeds of 1/1500 s, 1/1250 s, 1/800 s, 1/640 s, 1/500 s, 1/400 s, 1/250 s, 1/200 s and 1/100 s, respectively, achieved by adjusting the exposure compensation. Before photographs were taken, the background (sky) reference exposure was determined to be 1/1500 s (F4.2). The automatic exposure was 1/1500 s (E).

**Figure 3:**
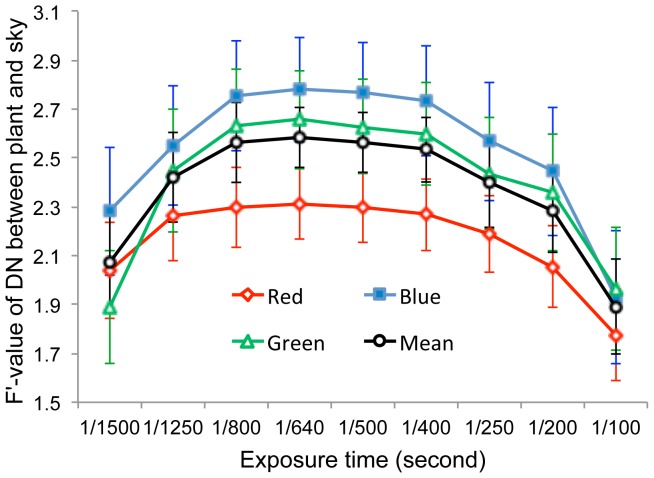
Variation in the F’ statistic for DN differences between background (sky) and vegetation pixels in upward-oriented photographs with exposures ranging from 1/1500 s to 1/100 s.

### 2.3 Non-destructive Determination of LAI and PAI

#### 2.3.1 Field photography

One month after the transplantation of *Potamogeton malainus*, we took vertical upward- and downward-oriented photographs to capture the vertical canopy cover fraction. The photographs were taken looking vertically both downward (with camera in the air above the water surface) and upward (with camera in the water) in order to compare the influence of photographic orientation on the ability to discriminate vegetation pixels from background pixels. We used an underwater camera (Pentax WG-2) with a resolution of 4608×3456 and a focal length of 37.1 mm (35 mm conversion) and saved the photographs in jpeg format. Both upward- and downward-oriented photographs were taken at a distance of 0.6 m to the water surface using a pole-pod with the camera mounted at one end. To ensure the camera was in the horizontal position needed for obtaining vertical photographs, we attached bubble-levels to the lens-side camera surface and to the opposite end of the pole-pod. Before taking photographs, we leveled the bubbles simultaneously. When taking photographs, we adjusted the pole-pod using the bubble level at the non-camera end to make certain the camera was horizontal. A single photo covered 0.56*0.42 m^2^, with the fields of view 50.0° (0°±25.0°) and 38.6° (0°±19.3°) in the length and width directions, respectively, of the incubator. The distance at which photographs were taken in this study (0.6 m to the water surface) was slightly less than previously recommended distances for terrestrial vegetation [10], [13]; this was to (1) reduce the distance subject to light penetration through the water and thus reduce possible interference caused by light absorption as well as the absorption, reflection and scattering of organic and inorganic particles in the water; and (2) cover as much as possible of the entire spatial footprints of the incubators by taking a total of 16 photographs above and below the water surface per incubator, thus ensuring the exact same coverage of aquatic vegetation was depicted in the photographs as was used in destructive measurement of LAI and PAI (we sampled all plants in the incubator to destructively measure LAI and PAI), which was difficult to achieve by increasing distance and decreasing photographs due to the height restriction of the incubators used in this study.

**Table 1:** Descriptive statistics, multiple comparisons and variance analysis of digital number (DN) between background (sky or bare sediment) and vegetation pixels for upward- and downward-oriented photographs.

			Min			Max			Means*			
	No.	Band	Background	Vegetation	F’	Background	Vegetation	F’	Background	Vegetation	Difference	F’
Up photo	136	Red	32.4	5.7	1.34	141.5	77.4	2.30	89.6 B	34.2 A	55.4 C	1.73 C
		Blue	37.7	6.4	1.90	161.7	79.4	3.10	98.0 B	30.7 A	67.3 CD	2.32 D
		Green	39.6	5.0	1.91	163.0	75.4	3.29	101.7 B	27.6 A	74.1 D	2.49 E
Down photo	108	Red	17.5	29.1	0.57	114.8	144.1	1.18	59.0 A	89.1 B	30.1 B	0.85 B
		Blue	14.4	22.9	0.57	120.6	146.4	1.10	58.0 A	83.6 B	25.6 B	0.83 B
		Green	13.1	15.9	0.21	132.0	148.2	0.69	60.4 A	71.6 B	11.2 A	0.46 A

* Means within columns followed by the same letter are not significantly different at 99% confidence level (Duncan’s Multiple Range Test).

To identify suitable light conditions, we took both upward- and downward-oriented photographs every two hours from 05:00 to 19:00 on a sunny day (28 May) to represent a wide range of sun zenith angles as well as conditions just before sunrise and after sunset (i.e. twilight periods). Automatic exposure was used when taking downward-oriented photographs, similar to the common practice for terrestrial vegetation [10], [13], [14]. Upward-oriented photographs were taken using a series of exposures obtained by adjusting the exposure compensation from −2.0 to 2.0 EV. Upward-oriented photographs in water without aquatic vegetation were taken to obtain a background (sky) reference exposure. To estimate the effect of water turbidity on the identification of vegetation pixels in both upward- and downward-oriented photographs, we artificially stirred the water in incubators with varying degrees of intensity to form a gradient of water turbidity and then took photographs and measured water turbidity simultaneously.

**Figure 4:**
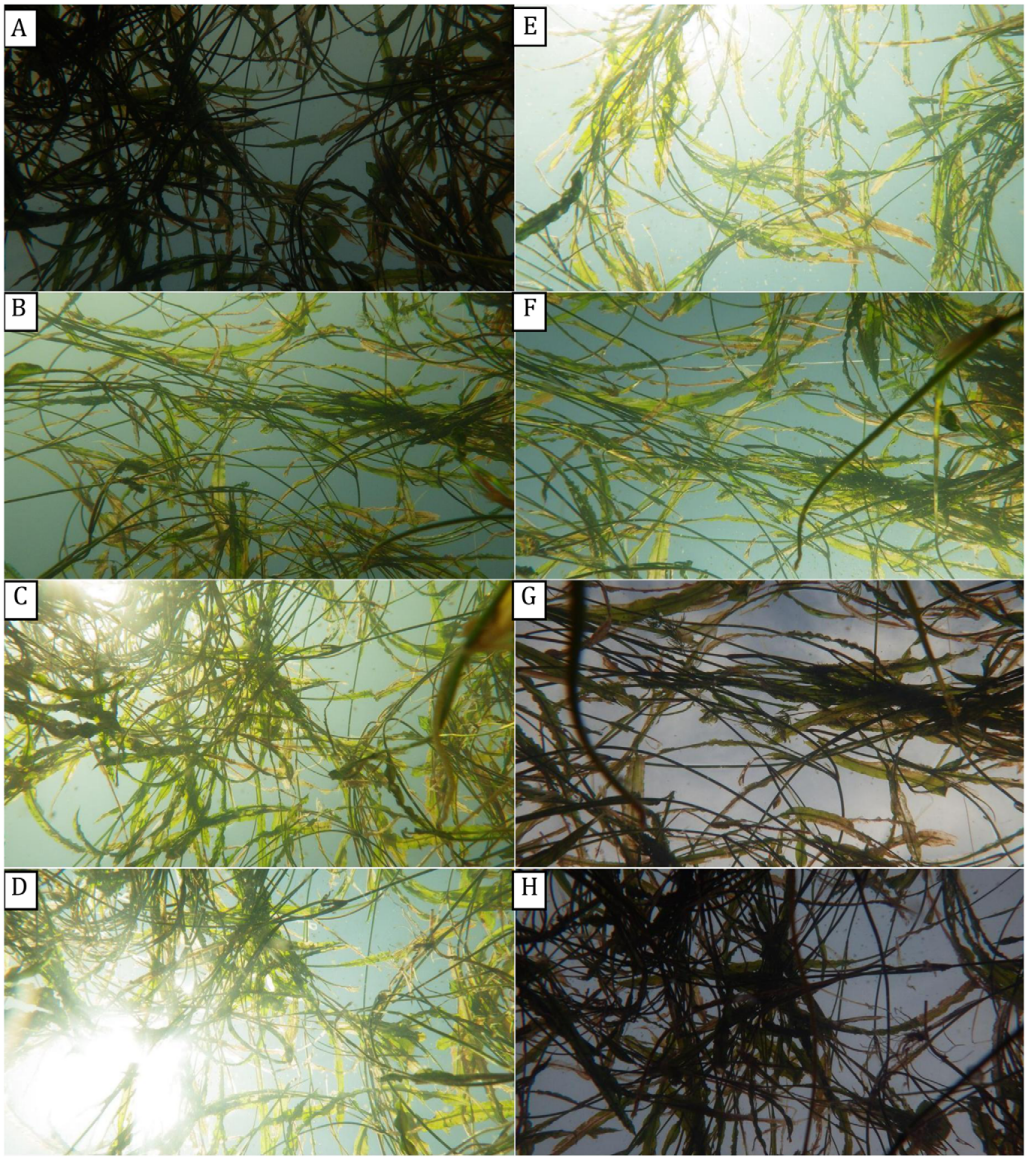
The influence of time of day on differences in digital number (DN) between vegetation and background pixels in vertical upward-oriented photographs. From A to H, the photographic times were 05:00, 07:00, 09:00, 11:00, 13:00, 15:00, 17:00, and 19:00 h, respectively. A and H were taken before sunrise and after sundown, respectively, when there was no direct sunlight.

**Figure 5:**
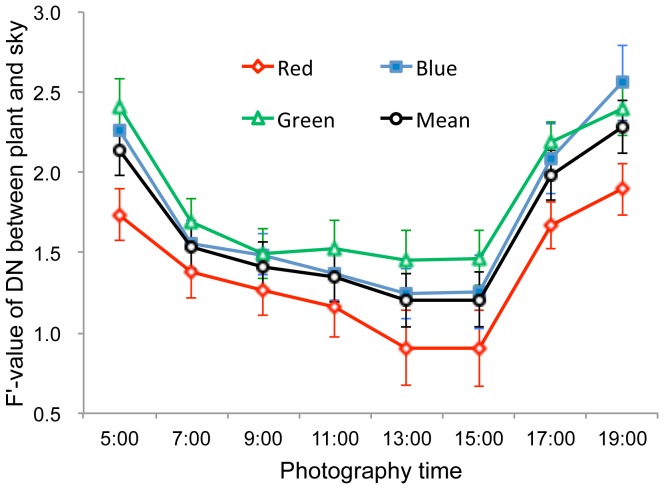
Variation in the F’ statistic for digital number (DN) differences between background (sky) and vegetation pixels with time of day ranging from before sunrise (05:00 h) to after sunset (19:00 h).

**Table 2:** Summary statistics for plant area index (PAI), leaf area index (LAI) and gap fraction in our study plots (i.e. incubators).

	Numbers	Min	Max	Mean	S.D.*
PAI	20	0.32	3.28	1.56	1.11
LAI	20	0.16	1.70	0.80	0.86
Gap fraction	20	0.17	0.81	0.47	0.51

* Standard deviation.

#### 2.3.2 Analytical methods for determination of optimal photographic parameters

Variance analysis was used to compare upward- and downward-oriented photographs and to determine the optimal light conditions and exposures for taking the photographs. All photographs were first classified as either vegetation or background using the Can-eye imaging freeware 6.2 (https://www4.paca.inra.fr/can-eye) with a binary classification (no mixed pixels, 2 classes) of plant (including both leaves and stems) and gap fraction. The F’-value was then calculated for every photograph to compare plant and background pixels.
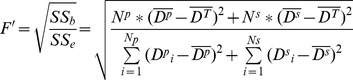
(1)


where SS_b_ and SS_e_ are sums of squared deviations between-group and within-group variance, respectively. *N^p^* and *N^s^* are numbers of plant and background pixels, respectively. 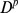 and 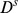 are the Digital Number (DN, i.e. scaled pixel intensity value) of plant and background pixels, respectively. 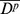, 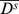 and 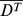 are the average DNs of plant, background and total pixels, respectively.

Finally, the F’-values were compared among photographs. Higher F’-values indicated a greater (i.e., more significant) difference in the DN between the background and vegetation pixels and thus a photograph that could be more easily classified. Our F’-value was independent of number of samples and thus could be used to compare the differences in plant and background pixels between photographs, which differs from the widely used F-value associated with Analysis of Variance (ANOVA).

**Figure 6:**
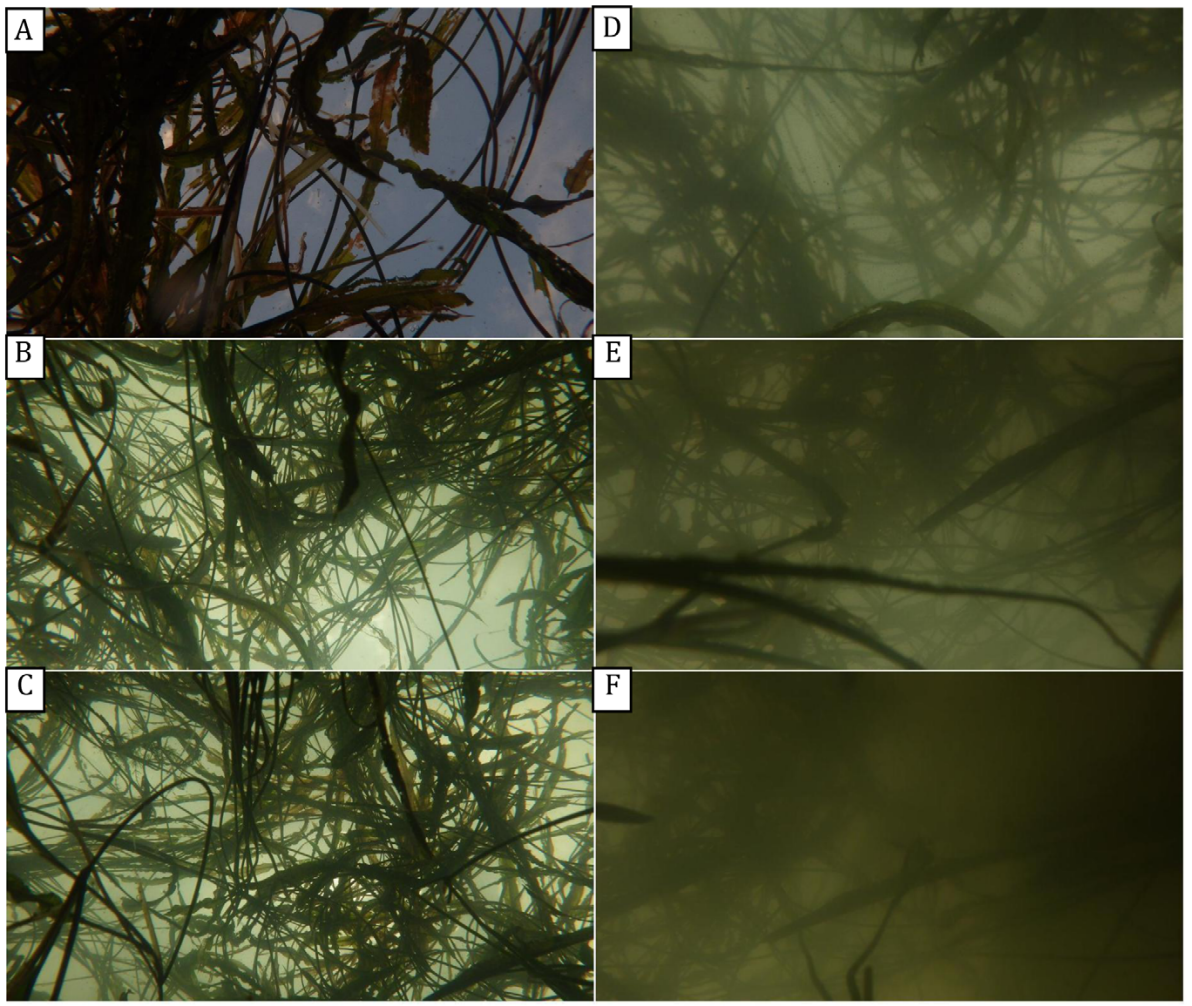
The influence of water turbidity on digital number (DN) differences between vegetation and background pixels in vertical upward-oriented photographs. From A to F, turbidity values were 2.0, 6.0, 10.0, 20.0, 25.0 and 30.0 NTU, respectively.

**Figure 7:**
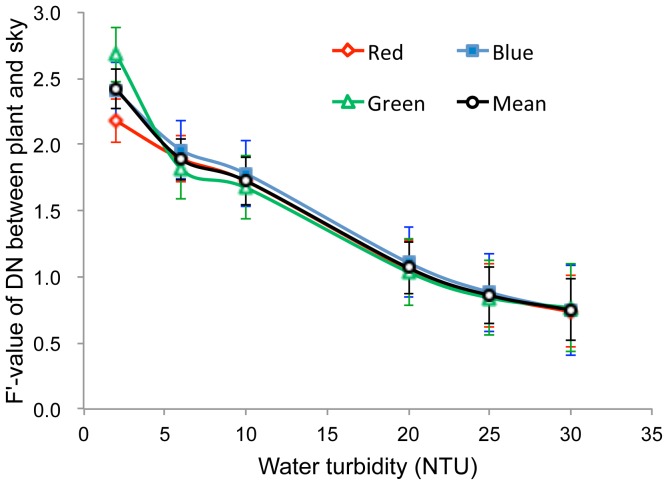
Variation in the F’ statistic for digital number (DN) differences between background (sky) and vegetation pixels in upward-oriented photographs with water turbidity ranging from 2.0 to 30.0 NTU.

#### 2.3.3 Determination of LAI and PAI using the gap fraction

Once the imaging software had calculated the gap fraction for all the field photographs, we examined the correlations between the derived vertical gap fraction and LAI and PAI and then compared the correlations with the modeled correlations using a Poisson model [12]. The foliage of a natural aquatic vegetation canopy could be considered to be azimuthally uniform and spatially random, and thus the Poisson model can be simplified as follows to describe the relationship between LAI and gap fraction [12], [13]:
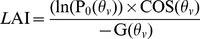
(2)


where θ_v_ is the zenith angle of the direction of the incident beam or the probe (viewer) penetrating the canopy, and 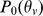 and 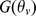 are canopy gap fraction and mean projection of a unit foliage area in the direction of θ_v_, respectively. Because of the difficulty of directly estimating the leaf inclination distribution function (LIDF) from the gap fraction measurement, the simplest (spherical) distribution model is generally sufficient [12]. In the spherical model, the average leaf inclination angle is 57.3°, and 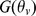 is nearly independent of θ_v_ which approximates 0.5. Therefore, for vertical photographs, LAI could be calculated using the following model [13]:
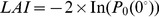
(3)


However, because each vertical photograph represents an area rather than a single point, the pixels in the vertical photographs obtained from our camera generally are not vertical, which influences the derivation of LAI [13], [14]. To evaluate the effect of the deviation from 0° of pixel angles in vertical photographs, we calculated the percentage ratio between the actual LAI (LAI_a_, i.e. calculated LAI using Equation (2), which uses the actual zenith angle of each pixel) and estimated LAI (LAI_e_, i.e., calculated LAI using Equation (3), in which all pixels are assumed to have an inclination angle of 0°):
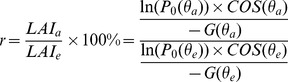
(4)


where P_0_(θ_a_) and P_0_(θ_e_) are the gap fraction at the inclination angles of θ_a_ and θ_e_, respectively. θ_a_ and θ_e_ are the actual inclination angle of a pixel and the “mistaken” inclination angle (i.e. 0°), respectively. For a real vertical photograph, the gap fraction is fixed and has nothing to do with whether we are “mistaking” its inclination angle, (i.e., P_0_(θ_a_) = P_0_(θ_e_)). In the spherical model describing the leaf inclination distribution function, 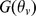 is nearly independent of θ_v_, approximating 0.5.; thus Equation (4) can be re-written as:
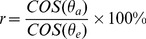
(5)


Using the parameters of our study (i.e., distance of 0.6 m to the water surface, resulting in photographic coverage of 0.56*0.42 m^2^), the calculation of LAI can be modified to:
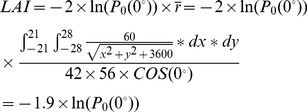
(6)


### 2.4 Destructive Determination of LAI and PAI

Immediately after photographs were taken, all plants in each incubator were harvested for destructive determination of LAI and PAI. Leaves and stems were separated. Approximately 20% of the leaves (or stems) from each incubator were placed on a 1×1 m^2^ glass sheet demarcated with tick marks and covered with another piece of transparent glass. Vertical photographs were then taken of the pressed leaves (stems) using a Pentax WG-2 camera. ERDAS IMAGINE 9.2 software was employed to make geometric corrections to the photographs according to the tick marks and to segregate each photograph into vegetative and background pixels, and thus the area of the leaves and the projected area of the stems were obtained. The pressed leaves (stems) were then placed into brown paper bags, dried to constant weight at 65°C and weighed, as were the remainder of the leaves (stems) harvested from the incubator. The total leaf (stem) area was calculated by multiplying leaf (stem) area per dry mass by the total dry mass in an incubator. LAI was then calculated as the ratio of leaf area to horizontal incubator area, and PAI was calculated as the ratio of the sum of leaf and stem area to horizontal incubator area.

**Figure 8:**
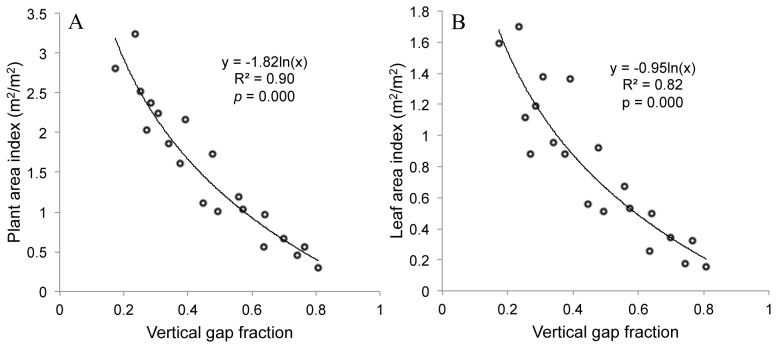
The relationship between vertical gap fraction calculated from upward-oriented photographs and plant area index (PAI, A), and leaf area index (LAI, B) calculated using a destructive harvesting approach.

## Results

### 3.1 Optimal Photographic Parameters for the Derivation of Gap Fraction

#### 3.1.1 Photographic direction

For all three bands (i.e., red, blue, and green), the DNs of background (i.e. sky) pixels were generally higher than those of aquatic vegetation pixels in the upward-oriented photographs, whereas the DNs of background (i.e. bare sediment) pixels were generally lower than those of aquatic vegetation for downward-oriented photographs (Fig. 1). Histograms of DN differed depending on photographic orientation as well, with upward-oriented photographs resulting in histograms with two peaks and downward-oriented photographs resulting in histograms with a single peak, suggesting that the difference in DN between vegetation and background was generally larger, and thus derivation of the gap fraction would be easier, in upward-oriented photographs relative to downward-oriented photographs. Descriptive statistics (Table 1) support the idea that the upward-oriented photographs were better suited to derivation of the gap fraction. Significantly larger differences in DN between the background and vegetation pixels were found in upward-oriented photographs than in downward-oriented photographs, with the average values for the red, blue, and green bands being 55.4, 67.3, and 74.1, respectively, for upward-oriented photographs and 30.1, 25.6, and 11.2, respectively, for downward-oriented photographs. Further, variance analysis indicated that F’-values between background and vegetation pixels in upward-oriented photographs were almost significantly higher than in downward-oriented photographs, with the average F’-values for red, blue and green bands being 1.73, 2.32 and 2.49 in upward-oriented photographs, respectively, and 0.85, 0.83 and 0.46 in downward-oriented photographs, respectively. Because our results all indicated that they were more suitable for the identification of vegetation pixels, and thus the derivation of gap fraction, upward-oriented photographs were used for the estimation of LAI using non-destructive means.

#### 3.1.2 Exposure setting

Using the exposure compensation function, we took upward-oriented photographs with shutter speeds varying from 1/1500 s to 1/100 s. Digital photographs taken with different exposures were visually different (Fig. 2). Lower exposures diminished the sharpness of the photographs and decreased the color, producing photographs that appeared similar to black-and-white photographs. F’ statistics describing the difference between sky and vegetation pixels gradually increased and then decreased with increasing exposure, peaking at 1/640 s (Fig. 3). ANOVA indicated no significant differences in F’-value for all three bands among photographs with exposures ranging from 1/1250 s to 1/400 s (*p* –values ranged from 0.16 to 0.83), with mean F’-values for the three bands varying between 2.42 and 2.58. These results clearly demonstrated that exposure settings could substantially influence the ability to distinguish vegetation from sky in upward-oriented photographs. Optimal performance was obtained at 1/640 s, but automatic exposure (1/500 s) exhibited only a very slight decrease in performance that was not likely to have a measurable impact on the results; therefore, automatic exposure seemed sufficient and was used in this study for distinguishing vegetation pixels and deriving the gap fraction for LAI estimation because of its operational convenience.

#### 3.1.3 Photographic time

Similar to the exposure trials, upward-oriented photographs differed visually according to time of day the photograph was taken (i.e., 05:00 to 19:00 hrs) (Fig. 4). Photographs taken before sunrise and after sundown (i.e. twilight periods without direct sunlight) were darker and more similar to black-and-white photographs than those taken at other times with direct, strong sunlight. Variance analysis resulted in F’-values that displayed single-valley curves for the red, blue and green bands from 05:00 h to 19:00 h (Fig. 5). Mean F’-values over all three bands decreased from 2.14 at 05:00 h to a low of 1.20 at 13:00 h, then increased to 2.28 at 19:00 h. Significantly higher mean F’-values of the three bands were observed in photographs taken at 05:00 h (before sunrise) and 19:00 h (after sundown) relative to photographs taken at other times (*p-*value ranging from 0.013 to 4.2*10^−8^). These findings suggested that photographs should be taken under diffuse light conditions such as before sunrise and after sundown to most easily distinguish vegetation pixels and derive the gap fraction from upward-oriented photographs.

#### 3.1.4 Water turbidity

Because the properties of water as a photographic medium can make it difficult to obtain clear photographs, we compared the upward-oriented photographs taken underwater at different levels of turbidity, finding that, as expected, more turbid waters resulted in lower photo clarity (Fig. 6). Variance analysis further confirmed this observation, with F’-values of the red, blue and green bands decreasing significantly with increasing water turbidity (Fig. 7, 0.001<*p*<0.002); the average of the three bands decreased from 2.42 to 1.07 as turbidity increased from 2 to 20 NTU. These results suggested that accurately distinguishing vegetation pixels from sky pixels becomes much more difficult when water turbidity is higher than 20 NTU.

### 3.2 Estimation of LAI and PAI using Vertical Gap Fraction

PAI, LAI and gap fraction varied widely among the incubators (Table 2), which allowed the results based on these data to be applied more universally. Leaf area accounted for an average of 51.1% of the total plant area, with the remaining 48.9% consisting of projected stem area. Because the projected stem area accounted for nearly half the total plant area, stems could not be ignored when calculating aquatic plant area in our study, and direct conversions of PAI into LAI may not be possible.

Significant logarithmic relationships existed between the vertical gap fraction derived from upward-oriented photographs and PAI and LAI calculated from destructive harvesting (Fig. 8), with R^2^ values of 0.90 and 0.82 for PAI and LAI, respectively (with corresponding *p*-values of 1.9*10^−10^ and 1.5*10^−7^, respectively). The coefficient of the model developed for the relationship between gap fraction and PAI (Fig. 8A) differed only slightly from that for the modified Poisson theoretical model in Equation (6) (coefficients were 1.82 and 1.9, respectively), indicating that vertical, upward-oriented photographs taken from underneath the water surface could be used to estimate PAI and LAI of the aquatic plant species *Potamogeton malainus* in our study.

## Discussion

### 4.1 Optimal Photographic Parameters

We found that the gap fraction could be obtained more easily from upward-oriented photographs than from downward-oriented photographs because of the greater distinction between vegetation and background. However, upward-oriented photography is probably only suitable for submerged vegetation with relatively long stems such as *Potamogeton malainus*, which was used in this study, as well as floating, floating-leaf and emergent vegetation, all of which are at or near the water surface. Upward-oriented photography may not be suitable for low submerged vegetation such as *Elodea nuttallii* in Taihu Lake, which is similar to terrestrial vegetation [9], [13].

An important issue related to the difference between upward- and downward-oriented photography should be noted here. For downward-oriented photographs, most of the stems and possibly some of the lower-layer leaves were not visible, resulting in the potential for underestimation of LAI. In upward-oriented photographs, however, LAI may have been overestimated because some stems were visible along with the leaves. Therefore, upward-oriented photography may be more suitable for the estimation of PAI rather than LAI [5], [10]. For the aquatic plant species examined in this study (i.e. *Potamogeton malainus*), projected stem area accounted for nearly half (48.9%) of the total plant area, suggesting that the effect of stems on the gap fraction derived from upward-oriented photographs could not be ignored and that PAI could not be considered a surrogate for LAI [10]. We also found a significant logarithmic relationship between the vertical gap fraction derived from upward-oriented photographs and LAI, which was likely due to the relatively stable relationship between PAI and LAI in different incubators since only one species was used in this study. Therefore, the vertical gap fraction derived from upward-oriented photographs may not be a sufficient predictor of LAI for aquatic vegetation communities composed of different species with different relationships between PAI and LAI.

For terrestrial vegetation, upward-oriented photographs should be taken under diffuse light conditions (e.g., cloudy days and twilight periods on sunny days) in order to ensure that the sensed radiation does not include any radiation reflected or transmitted by leaves [8]. As for exposure, both automatic exposure and exposure that is two levels higher than open sky exposure have been recommended for upward photography [10], [31]. Despite the effects on photographs of light absorption by water as well as suspension of organic and inorganic particles, optimal photographic parameters similar to those recommended for terrestrial vegetation were found for aquatic vegetation in this study. Our results indicated that upward-oriented photographs of aquatic vegetation should be taken under diffuse light conditions such as before sunrise or after sundown for best calculating the gap fraction. Optimal exposure in our study was 1/640 s when the background (sky) reference exposure was 1/1500 s. Automatic exposure achieved very nearly the same performance and was thus recommended because of its operational convenience.

Although the atmospheric medium is typically not a major consideration when photographing terrestrial vegetation, the properties of water as a medium need to be addressed for photography of aquatic vegetation. Strong light absorption and strong light reflection off organic and inorganic particles make water less transparent than air. We found that water turbidity greater than 20 NTU made distinguishing vegetation from sky in the photographs difficult. Moreover, this restriction could not be reduced by decreasing the distance between the camera and water surface because too short a distance could also decrease the accuracy of the LAI measurement from the gap fraction by not only capturing a less-representative spatial coverage but also by having individual leaves too close to the sensor [10], [13]. Fortunately, because of the purification function of aquatic vegetation as well as the stringent water quality requirements of submerged vegetation, submerged vegetation can only exist in relatively clear water with turbidity lower than 20 NTU [32]–[39], which makes our method feasible in most cases.

### 4.2 Relationship between Vertical Gap Fraction and PAI

Similar to terrestrial vegetation [12]–[14], an exponential function was found to be appropriate for describing the relationship between gap fraction and PAI (Fig. 8). The coefficients of the model we developed in Fig. 8A (1.82) and the modified Poisson theoretical model depicted in Equation (6) (1.9) were only slightly different. The difference in the coefficients can likely be explained from two perspectives. Firstly, one of the assumptions of Equation (6) was that the spherical model was a good descriptor of the leaf inclination distribution function for the aquatic vegetation examined in this study (i.e., 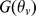 = 0.5 in Equation (3)) [12]. However, for *Potamogeton malainus* plants, most stems (accounting for nearly half of PAI) are nearly vertical while most leaves are nearly flat. It is likely that none of the models in current and widespread use for describing the leaf inclination distribution function for terrestrial plants, including the spherical model used in this study, are sufficient for describing the morphology of *Potamogeton malainus* [10], [12], [40]–[42]. Secondly, Equation (6) also required that the canopy foliage be completely randomly distributed, and the ratio of the coefficients between models using destructive data and the theoretical models was specified to be a clumping coefficient. For some types of canopy such as row crops, the clumping effect generally needs to be estimated [12], [14]. Similar to row crops, *Potamogeton malainus* in our study was transplanted in incubators in rows and thus a clumping effect probably existed, although this effect was likely insignificant.

Nevertheless, the model we developed using gap fraction derived from upward-oriented photographs and destructive measurement of PAI (Fig. 8A) is very similar to the modified Poisson model of Equation (6), further confirming the feasibility of using vertical photographs for estimating PAI of aquatic vegetation. To the best of our knowledge, this is the first study that has developed a non-destructive approach to measurement of PAI and LAI of aquatic vegetation using digital cameras.
